# Dutch nOcturnal and hoME dialysis Study To Improve Clinical Outcomes (DOMESTICO): rationale and design

**DOI:** 10.1186/s12882-019-1526-4

**Published:** 2019-09-18

**Authors:** A. van Eck van der Sluijs, A. A. Bonenkamp, F. W. Dekker, A. C. Abrahams, B. C. van Jaarsveld, on behalf of the DOMESTICO study group, A. C. Abrahams, B. C. van Jaarsveld, F. W. Dekker, A. van Eck van der Sluijs, A. A. Bonenkamp, M. C. Verhaar, F. J. van Ittersum, J. A. J. Bart, M. H. Hemmelder, E. L. Penne, D. G. Struijk, A. Özyilmaz, M. M. Versteegh, L. Hakkaart-van Roijen, G. A. de Wit, F. T. Boereboom, T. A. Kanters, G. de Graaf, P. Leurs, M. R. Korte, A. M. Schrander, T. T. Cnossen, J. Lips, H. P. Krepel, M. A. G. J. ten Dam, C. J. A. M. Konings, C. J. Doorenbos, A. Lips, F. T. J. Boereboom, S. van Esch, C. R. Susanto, G. F. van Breda, E. J. Hoorn, D. Severs, A. H. Boonstra, R. W. Nette, Y. M. Vermeeren, D. H. T. Ijpelaar, H. D. Thang, N. H. Hommes, M. van Buren, J. M. Hofstra, S. H. A. Diepeveen, E. K. Hoogeveen, T. Cornelis, S. Boorsma, J. I. Rotmans, A. M. van Alphen, F. van der Sande, E. J. R. Litjens, W. M. T. Janssen, A. Kuijper, C. H. Beerenhout, H. S. Brink, R. Wijering, R. J. Bosma, C. W. H. de Fijter, H. F. H. Brulez, H. W. van Hamersvelt, S. J. Huisman, M. P. Kooistra, J. C. Verhave, G. van Kempen, H. Klein, C. E. Douma, H. H. Vincent, W. J. W. Bos, J. D. Snoep, J. Mulder, C. F. M. Franssen, A. J. Luik, R. J. L. Klaassen, A. G. Weenink, M. M. E. Krekels

**Affiliations:** 1 Department of Nephrology and Hypertension, University Medical Center Utrecht, Utrecht, The Netherlands; 2 Department of Nephrology, Amsterdam University Medical Centers, Vrije Universiteit Amsterdam, Amsterdam, The Netherlands; 3 Department of Clinical Epidemiology, Leiden University Medical Center, Leiden, The Netherlands; 4 Diapriva Dialysis Center, Amsterdam, The Netherlands

**Keywords:** Clinical outcomes, Costs, Health-related quality of life, Home dialysis, Home haemodialysis, In-Centre haemodialysis, Peritoneal dialysis

## Abstract

**Background:**

More than 6200 End Stage Renal Disease patients in the Netherlands are dependent on dialysis, either performed at home or in a dialysis centre. Visiting a dialysis centre three times a week is considered a large burden by many patients. However, recent data regarding the effects of dialysis at home on quality of life, clinical outcomes, and costs compared with in-centre haemodialysis are lacking.

**Methods:**

The Dutch nOcturnal and hoME dialysis Study To Improve Clinical Outcomes (DOMESTICO) is a nationwide, prospective, observational cohort study that will include adult patients starting with a form of dialysis. Health-related quality of life, as the primary outcome, clinical outcomes and costs, as secondary outcomes, will be measured every 3–6 months in patients on home dialysis, and compared with a control group consisting of in-centre haemodialysis patients. During a 3-year period 800 home dialysis patients (600 peritoneal dialysis and 200 home haemodialysis patients) and a comparison group of 800 in-centre haemodialysis patients will be included from 53 Dutch dialysis centres (covering 96% of Dutch centres) and 1 Belgian dialysis centre (covering 4% of Flemish centres).

**Discussion:**

DOMESTICO will prospectively investigate the effect of home dialysis therapies on health-related quality of life, clinical outcomes and costs, in comparison with in-centre haemodialysis. The findings of this study are expected to ameliorate the shared decision-making process and give more guidance to healthcare professionals, in particular to assess which type of patients may benefit most from home dialysis.

**Trial registration:**

The DOMESTICO study is registered with the National Trial Register on (number: NL6519, date of registration: 22 August 2017) and the Central Committee on Research Involving Human Subjects (CCMO) (number: NL63277.029.17).

**Electronic supplementary material:**

The online version of this article (10.1186/s12882-019-1526-4) contains supplementary material, which is available to authorized users.

## Background

In the Netherlands, over 6200 patients with End Stage Renal Disease (ESRD) are dependent on dialysis, and over the past 15 years, the number of dialysis patients has increased by more than 20% [1–3]. The burden of dialysis is high and the health-related quality of life (HRQoL), which is presently considered to be the most important outcome parameter in dialysis patients, is much worse than that of healthy people [4]. As patient survival is poor, with a median five-year survival rate of only 45%, optimising HRQoL is of great importance for this growing group of patients [5, 6].

Besides its impact on HRQoL, dialysis is also an expensive treatment. In the Netherlands, the estimated costs are approximately 570 million euro per year (639 million US dollars) and are still increasing. [Personal communications, G.A. De Wit, National Institute for Public Health and the Environment, 2019] This makes dialysis by far the highest cost-consuming treatment in internal medicine, not only calculated per individual patient, but also if total treatment costs are taken into account [7].

Home dialysis has a potential positive effect on HRQoL because it offers flexibility to patients and greater freedom [8]. Moreover, home dialysis is possibly a more cost-effective therapy if less nursing staff is needed, when patients perform their treatment autonomously or with help of an informal caregiver. Despite these potential advantages, currently more than 80% of dialysis patients are treated with in-centre haemodialysis (ICHD). Furthermore, the percentage of patients treated with home dialysis is steadily decreasing in the Netherlands, from 32% in 2002 to 18% in 2018. This decline is mainly attributable to a reduction in the number of patients performing peritoneal dialysis (PD), the main home based therapy, with 1519 PD patients (30% of total dialysis patients) in 2002 versus 894 PD patients (14% of total dialysis patients) in 2018 [1].

Available evidence regarding the effects of home dialysis compared with ICHD on HRQoL, a Patient Reported Outcome (PRO), is limited. Most studies have a cross-sectional design and lack adequate correction for confounding among dialysis groups [9–38]. Also, the characteristics of patients starting with some kind of home dialysis treatment have changed remarkably over the past years. Previously, those patients were typically young, working people with little comorbidities, whereas during the last years the general home dialysis population is older and often suffers from multiple comorbidities [2]. This could influence clinical outcomes such as mortality and hospitalisation rate. Finally, there are limited data available regarding the cost-effectiveness of home dialysis.

To investigate the effect of home dialysis on HRQoL, clinical outcomes, and costs, the Dutch nOcturnal and hoME dialysis Study To Improve Clinical Outcomes (DOMESTICO) has been initiated. The aim of this study is to compare HRQoL, clinical outcomes, and cost-effectiveness of home dialysis with ICHD. The hypothesis is that home dialysis is associated with better HRQoL, at least comparable clinical outcomes and lower costs, compared to ICHD.

## Methods

### Study design

**Fig. 1:**
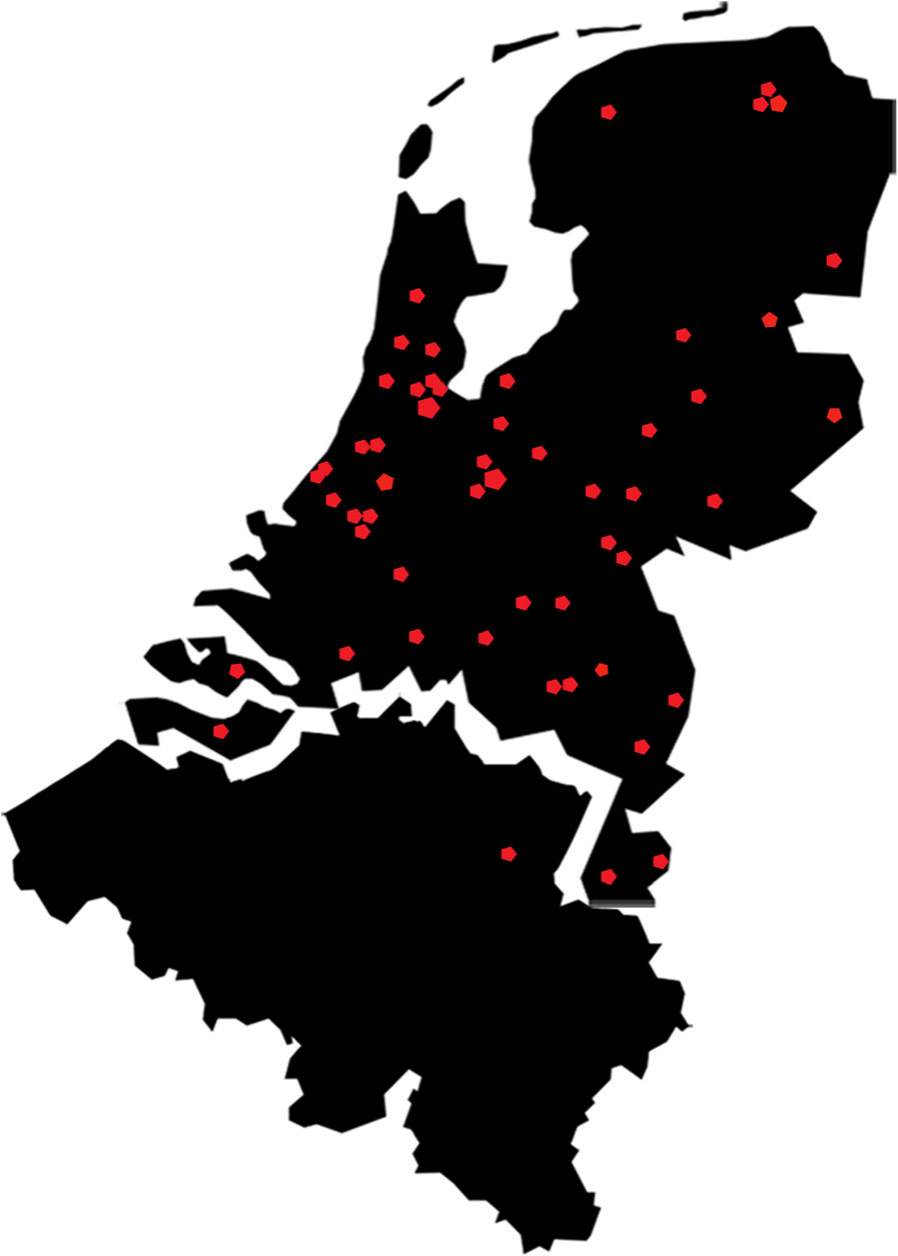
Participating centres. The red dots indicate the participating centres: 53 Dutch dialysis centres (covering 96% of Dutch centres) and 1 Belgian dialysis centre

DOMESTICO is a nationwide, prospective, observational cohort study comparing home dialysis with ICHD. The maximum follow-up period of the study is 48 months. At present, 53 Dutch dialysis centres (covering 96% of Dutch centres) and 1 Belgian dialysis centre have agreed to recruit patients (Fig. 1). The study is conducted according to the principles of the Declaration of Helsinki and in accordance with the Medical Research Involving Human Subjects Act (WMO).

### Study population

All patients, aged 18 years and older, with ESRD that start with a form of dialysis in the participating centres, between December 2017 and December 2020, are eligible for this study. These patients are allowed to have a history of renal replacement therapy (RRT), however they have to (re) start dialysis during the study period for example due to kidney transplant failure (with or without previous dialysis). All these patients are defined as ‘incident patients’. Prevalent dialysis patients, and patients with a life expectancy shorter than 3 months or an expected kidney transplantation within 3 months, are excluded. Patients have to provide written informed consent before participating in the study.

### Inclusion

Patients are included in the period within four weeks before to four weeks after start of dialysis. If patients are missed for inclusion within this timeframe (for example, due to acute start of dialysis), they can be included at 3 months (± 2 weeks) after start of dialysis. Start of dialysis is defined as the first PD session performed at (a nursing) home (excluding PD-training) or, in case of ICHD, the first haemodialysis session performed in a centre (excluding continuous RRT).

**Fig. 2:**
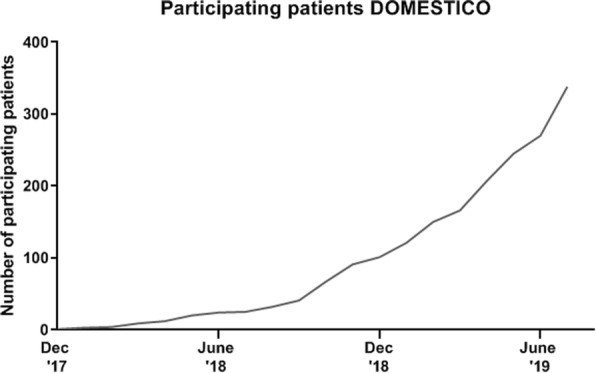
Participating patients

The first patient was included in December 2017 and the study has currently started in 45 centres with 338 participating patients (Fig. 2).

### (Early) termination

For each participating patient, the study ends on 20 December 2021. Early study termination occurs if the patient withdraws from the study or stops dialysis treatment. Reasons to stop dialysis include kidney transplantation, recovery of kidney function, the wish to stop dialysis, or death.

### Outcomes

#### Primary outcome parameter

The primary outcome parameter is the patient’s HRQoL, a PRO, determined with the 12-item Short Form (SF-12) health survey and the Dialysis Symptom Index (DSI) [39, 40]. These questionnaires were carefully selected as Patient Reported Outcome Measures (PROMs) in nephrological care by the Dutch Kidney Patients Association, the Dutch Federation for Nephrology, Nefrovisie (the Dutch Quality Institute for Nephrology), and Leiden University Medical Center [41, 42].

The SF-12 is the shorter version of the Short Form-36 (SF-36), one of the most widely used surveys to assess HRQoL [43, 44]. The SF-36 consists of eight domains: Physical functioning, Role-physical, Bodily pain, General health, Vitality, Social function, Role-emotional and Mental health. These domains are summarised in the Physical Component Summary (PCS) score and the Mental Component Summary (MCS) score. In the SF-12 these summary scores are calculated from the 12 most important questions (explaining ~ 90% variance) of the SF-36 questionnaire [39, 45]. As the average difference in summary scores between SF-36 and SF-12 is quite small, for time-efficiency reasons, the SF-12 can be used reliably in cohort studies [46].

The DSI consists of 30 questions evaluating the severity of symptoms relevant to dialysis and ESRD patients (Table 1). Patients report the level of burden of specific symptoms on a 5-point Likert scale, options range from ‘not at all bothersome’ to ‘very bothersome’ [40].

**Table 1:** Items Dialysis Symptom Index

1. Constipation	16. Chest pain
2. Nausea	17. Headache
3. Vomiting	18. Muscle soreness
4. Diarrhoea	19. Difficulty concentrating
5. Decreased appetite	20. Dry skin
6. Muscle cramps	21. Itching
7. Swelling in legs	22. Worrying
8. Shortness of breath	23. Feeling nervous
9. Lightheadedness or dizziness	24. Trouble falling asleep
10. Restless legs or difficulty keeping legs still	25. Trouble staying asleep
11. Numbness or tingling in feet	26. Feeling irritable
12. Feeling tired or lack of energy	27. Feeling sad
13. Cough	28. Feeling anxious
14. Dry mouth	29. Decreased interest in sex
15. Bone or joint pain	30. Difficulty becoming sexually aroused

#### Secondary outcome parameters

Secondary outcome parameters are hospitalisation, mortality, other clinical parameters, costs, and technique failure.

The cause of each hospitalisation episode will be categorised into the following categories (using ICD-10 codes) [47]:

- Cardiac (including myocardial ischaemia/infarction, cardiac arrest/arrhythmia, cardiac failure, fluid overload/pulmonary edema, haemorrhagic pericarditis);
- Vascular disease (including pulmonary embolus, stroke, cerebrovascular haemorrhage, ruptured vascular aneurysm, mesenteric infarction, peripheral arterial disease);
- Infection, non-dialysis related (including bacteraemia/sepsis, cardiac infection, HIV, osteomyelitis, respiratory infection, urinary tract infection);
- Dialysis related (including dialysis access infection, peritonitis, PD catheter leakage/exchange/removal, fistula operation, renal fluid overload, bleeding);
- Malignancy;
- Bleeding, non-dialysis related (including intracranial bleeding, gastro-intestinal bleeding, other causes of bleeding);
- Other causes.

Mortality will be categorised into the following categories (using ERA-EDTA codes) [48]:

- Sudden death ‘with unknown cause’;
- Cardiac (including myocardial ischaemia/infarction, cardiac arrest/arrhythmia, cardiac failure, fluid overload/pulmonary edema, haemorrhagic pericarditis);
- Vascular (including pulmonary embolus, stroke, cerebrovascular haemorrhage, ruptured vascular aneurysm, mesenteric infarction, peripheral arterial disease);
- Infectious, dialysis related (including dialysis access infection, peritonitis);
- Infectious, non-dialysis related (including bacteraemia/sepsis, cardiac infection, HIV, osteomyelitis, respiratory infection, urinary tract infection);
- Malignancy;
- Bleeding (including dialysis related bleeding, intracranial bleeding, gastro-intestinal bleeding, other causes of bleeding);
- Overall deterioration in clinical condition/stopping dialysis;
- Other causes.

Besides hospitalisation and mortality, several clinical parameters will be recorded including blood pressure and use of antihypertensive drugs, haemoglobin and use of erythropoiesis-stimulating agents, phosphate levels and use of phosphate binders, vascular access parameters, and nutritional status.

Direct healthcare costs, patient costs, and costs with regard to productivity losses will be assessed with a subset of questions from the Institute for Medical Technology Assessment (iMTA) Productivity Cost Questionnaire (iPCQ) and the iMTA Medical Cost Questionnaire (iMCQ) [49, 50]. To capture all health care costs for the population under research a small number of disease specific services are added to the standard iMCQ, e.g. home dialysis. Given the fact that many patients need substantial help from close relatives, also use of informal care by patients will be assessed. The costs related to the healthcare consumption, the dialysis procedures, the diagnostic tests and (over-the-counter) medication will be derived from the patient’s medical chart during the study. Unit costs will be derived from the Dutch manual for costing studies [51].

To further examine cost-effectiveness, the EuroQol-5D-5L (EQ5D-5L) questionnaire will be used. The EQ-5D-5L measures HRQoL on the following 5 domains: mobility, self-care, usual activities, pain/discomfort, and anxiety/depression. Each domain has 5 levels of functioning, ranging from ‘no problems’ to ‘extreme problems’. The EQ-5D-5L also contains a visual analogue scale on which the current health state can be indicated. The EQ-5D scores can be used to calculate utilities, which describe HRQoL on a scale from 0 (dead) to 1 (perfect health). Utilities can be combined with survival to calculate quality adjusted life years (QALYs). As outcome measure for cost-effectiveness, the costs per additional QALY will be analysed [52, 53].

All participating patients will also receive a self-management screening questionnaire (SeMaS) at baseline, in order to investigate whether self-management can predict a successful home dialysis treatment. This questionnaire shows the abilities and possible barriers for self-management by asking questions about the burden of disease, locus of control, self-efficacy, social support, coping style, anxiety, depression, and skills [54, 55].

Table 2 provides an overview of the moments when participating patients will fill in the aforementioned questionnaires.

**Table 2:** Overview questionnaires

Visit	SF-12 and DSI	iPCQ and iMCQ	EQ-5D-5L	SeMaS
Baseline	X	X	X	X
At 3 and 6 months	X	X	X	
At 9 months and every 6 months thereafter		X		
At 12 months and every 6 months thereafter	X	X	X	

*SF-12* Short Form-12, *DSI* Dialysis Symptom Index, *iPCQ* Institute for Medical Technology Assessment (iMTA) Productivity Cost Questionnaire, *iMCQ* iMTA Medical Cost Questionnaire, *SeMaS* self-management screening questionnaire

Finally, technique failure rate of home dialysis, defined by a composite outcome of death or transfer to ICHD, will be assessed. Both a 30-days and a 180-days definition of technique failure will be used according to the minimum number of days the patient received ICHD after cessation of home dialysis [56]. Permanent technique failure is defined by death or transfer to ICHD (using the 180-days definition), or cessation of dialysis. Death-censored technique failure will be reported separately. Transfer to kidney transplantation is not considered to be technique failure and will also be reported separately [56].

### Data collection

All study outcomes, except the SeMaS, will be assessed at baseline, after 3 months, 6 months, and thereafter every 6 months until end of follow-up or end of the study (Table 2).

Data will be registered in case report forms (CRF). IBM Data Collection will be used as CRF. The database is developed by Nefrovisie and follows the principles of Good Clinical Practice (i.e. it has an audit trail, possibility for electronic signing, direct validation of inserted data, authorisation per form and user). Nefrovisie will also host the database for the duration of the study. The database will be archived for future research during 15 years after termination of the study.

### Statistical analysis

All statistical analyses will be performed using statistical software such as SPSS and Stata. Univariable and multivariable regression analysis will be conducted. In case of repeated measures, multilevel analysis or generalised estimating equations will be applied. Possible confounders determined a priori are age, gender, marital status, level of education, work status, cause of renal failure, prior RRT with dialysis vintage, comorbidities, albumin, body mass index, and protein energy wasting. Cumulative incidence of hospitalisation, mortality, and technique failure will be reported in Kaplan Meier curves. In case of missing data, multiple imputation techniques will be used to impute the missing values where appropriate.

Overall costs will be compared across the treatment groups and 95% confidence intervals will be estimated using bootstrapping techniques. The cost-effectiveness of different dialysis modalities will be determined using a state transition model. This model captures the changes in treatment modality, including transplantation, over time. The results of the DOMESTICO study will be used as input parameters for this model.

### Sample size calculation

For the primary outcome HRQoL, obtained with the SF-12, a sample size of 350 patients is required. To obtain a clinically relevant difference between groups of 3 points in the SF-12 summary scores, after a median of 12 months follow-up, 175 patients per group are needed (assumed standard deviation = 10 points, α = 0.05, β = 0.20) [46, 57–59].

However, for the EQ5D-5L, an important component for the secondary outcome cost-effectiveness, a sample size of 1400 patients (700 patients per group) is needed. A difference of 0.03–0.07 points between groups after a mean follow-up of 12 months is considered clinically relevant [44, 60, 61]. The standard deviation in dialysis groups ranges from 0.1 to 0.22 [62, 63]. Assuming a common standard deviation of 0.20 and the lowest, still clinically relevant score, a total of 1400 patients (700 patients per group) will be sufficient to detect a difference of 0.03 points in the EQ5D-5L score between groups (α = 0.05, β = 0.20).

When approximately 10% loss to follow up is taken into account, a group of 800 home dialysis patients and a comparison group of 800 ICHD patients has to be included in order to have sufficient power to analyse both outcomes. Since the ratio between PD patients and home haemodialysis (HHD) patients in the Netherlands is expected to be 3:1 in future years, the home dialysis group will consist of 600 PD and 200 HHD patients.

## Discussion

Dialysis has a great impact on the HRQoL of ESRD patients and dialysis is a very expensive treatment. More than 80% of Dutch dialysis patients are treated with ICHD although home dialysis could result in a better HRQoL and could be more cost effective. Therefore, we initiated the DOMESTICO study, which will investigate the effects of home dialysis on HRQoL in relation to clinical outcomes and costs, in comparison with ICHD. This nationwide cohort study will include 1600 incident dialysis patients over a period of 3 years. At time of submission of this manuscript, 338 patients have been included.

Although a randomised controlled trial (RCT) would yield the ultimate answer to our research question, this is not in accordance with the concept of shared decision making. A patient’s choice between home dialysis and ICHD is considered too fundamental, to let it be determined by chance. Indeed, an RCT in the Netherlands comparing PD with ICHD conducted in the past, stopped early due to poor patient recruitment; only 38 patients consented to be randomly assigned to either PD or ICHD [64]. Hence, DOMESTICO is designed as a prospective, observational cohort study collecting extensive parameters to correct for confounding by indication.

The results of this study will be of great importance for future ESRD patients when choosing a treatment, as HRQoL is increasingly acknowledged by clinicians and patients as an important aspect in the decision-making process. In addition, the results with respect to clinical outcomes will ameliorate the shared decision-making process. Finally, the data could give more guidance to healthcare professionals, in particular to assess which type of patients may benefit most from home dialysis.

## Additional file

Additional file 1: Local ethics committees/IRBs DOMESTICO. This file contains a list of the 44 (out of 53) local ethics committees from which approval for DOMESTICO is obtained. (DOCX 17 kb)

## Data Availability

Not applicable.

## Abbreviations

CCMO: Central Committee on Research Involving Human Subjects
CRF: Case report forms
DOMESTICO: Dutch nOcturnal and hoME dialysis Study To Improve Clinical Outcomes
DSI: Dialysis Symptom Index
EQ5D-5L: EuroQol-5D-5L
ESRD: End Stage Renal Disease
HHD: Home haemodialysis
HRQoL: Health-related quality of life
ICHD: In-centre haemodialysis
iMCQ: iMTA Medical Cost Questionnaire
iMTA: Institute for Medical Technology Assessment
iPCQ: iMTA Productivity Cost Questionnaire
MCS: Mental Component Summary
PCS: Physical Component Summary
PD: Peritoneal dialysis
PRO: Patient Reported Outcome
PROM: Patient Reported Outcome Measure
QALY: Quality adjusted life year
RCT: Randomised controlled trial
RRT: Renal replacement therapy
SeMaS: Self-management screening questionnaire
SF: Short Form

## References

[1] Nefrovisie. http://www.nefrovisie.nl/nefrodata/. Accessed 20 June 2019.

[2] Hemke AC, Dekker FW, Bos WJW (2012). Oorzaken voor verminderd aandeel peritoneale dialyse als nierfunctievervangende behandeling in Nederland. Ned Tijdschr Geneeskd.

[3] Volksgezondheidenzorg.info. https://www.volksgezondheidenzorg.info/. Accessed 2 May 2019.

[4] Gorodetskaya I, Zenios S, McCulloch CE (2005). Health-related quality of life and estimates of utility in chronic kidney disease. Kidney Int.

[5] van de Luijtgaarden MWM, Jager KJ, Segelmark M (2016). Trends in dialysis modality choice and related patient survival in the ERA-EDTA registry over a 20-year period. Nephrol Dial Transplant.

[6] Kramer A, Pippias M, Noordzij M (2018). The European renal association - European Dialysis and transplant association (ERA-EDTA) registry annual report 2015: a summary. Clin Kidney J.

[7] Nederlandse Zorgautoriteit. http://opendisdata.nl/. Accessed 2 May 2019.

[8] Ageborg M, Allenius BL, Cederfjall C (2005). Quality of life, self-care ability, and sense of coherence in hemodialysis patients: a comparative study. Hemodial Int.

[9] Al Wakeel J, Al Harbi A, Bayoumi M (2012). Quality of life in hemodialysis and peritoneal dialysis patients in Saudi Arabia. Ann Saudi Med.

[10] Atapour A, Nasr S, Boroujeni AM (2016). A comparison of the quality of life of the patients undergoing hemodialysis versus peritoneal dialysis and its correlation to the quality of dialysis. Saudi J Kidney Dis Transpl..

[11] Barata NE (2015). Dyadic relationship and quality of life patients with chronic kidney disease. J Bras Nefrol.

[12] Basok EK, Atsu N, Rifaioglu MM (2009). Assessment of female sexual function and quality of life in predialysis, peritoneal dialysis, hemodialysis, and renal transplant patients. Int Urol Nephrol.

[13] Baykan H, Yargic I (2012). Depression, anxiety disorders, quality of life and stress coping strategies in hemodialysis and continuous ambulatory peritoneal dialysis patients. Klinik Psikofarmakoloji Bulteni.

[14] Borowiak E, Braksator E, Nowicki M (2009). Quality of life of chronic hemodialysis and peritoneal dialysis patients. Clin Exp Med Lett.

[15] Brown EA, Johansson L, Farrington K (2010). Broadening options for long-term Dialysis in the elderly (BOLDE): differences in quality of life on peritoneal dialysis compared to haemodialysis for older patients. Nephrol Dial Transplant.

[16] Chen JY, Wan EYF, Choi EPH, et al. The health-related quality of life of Chinese patients on hemodialysis and peritoneal Dialysis. Patient. 2017:1–10. 10.1007/s40271-017-0256-6 28589314

[17] Czyzewski L, Sanko-Resmer J, Wyzgal J (2014). Assessment of health-related quality of life of patients after kidney transplantation in comparison with hemodialysis and peritoneal dialysis. Ann Transplant.

[18] Fructuoso M, Castro R, Oliveira L (2011). Quality of life in chronic kidney disease. Nefrologia..

[19] Goncalves FA, Dalosso IF, Borba JM (2015). Quality of life in chronic renal patients on hemodialysis or peritoneal dialysis: a comparative study in a referral service of Curitiba - PR. Jornal brasileiro de nefrologia.

[20] Ibrahim N, Chiew-Tong NK, Desa A (2011). Symptoms and health-related quality of life in patients with heamodialysis and continuous ambulatory peritoneal dialysis. Res J Med Sci.

[21] Ikonomou M, Skapinakis P, Balafa O (2015). The impact of socioeconomic factors on quality of life of patients with chronic kidney disease in Greece. J Ren Care.

[22] Kim JY, Kim B, Park KS (2013). Health-related quality of life with KDQOL-36 and its association with self-efficacy and treatment satisfaction in Korean dialysis patients. Qual Life Res.

[23] Kontodimopoulos N, Pappa E, Niakas D (2009). Gender- and age-related benefit of renal replacement therapy on health-related quality of life. Scand J Caring Sci.

[24] Liu WJ, Musa R, Chew TF (2014). Quality of life in dialysis: a Malaysian perspective. Hemodial Int.

[25] Maglakelidze N, Pantsulaia T, Tchokhonelidze I (2011). Assessment of health-related quality of life in renal transplant recipients and dialysis patients. Transplant Proc.

[26] Nakayama M, Ishida M, Ogihara M (2015). Social functioning and socioeconomic changes after introduction of regular dialysis treatment and impact of dialysis modality: a multi-Centre survey of Japanese patients. Nephrology (Carlton).

[27] Okpechi IG, Nthite T, Swanepoel CR (2013). Health-related quality of life in patients on hemodialysis and peritoneal dialysis. Saudi J Kidney Dis Transpl.

[28] Ören B, Enc N (2013). Quality of life in chronic haemodialysis and peritoneal dialysis patients in Turkey and related factors. Int J Nurs Pract.

[29] Ramos EC, Santos I, Zanini R (2015). Quality of life of chronic renal patients in peritoneal dialysis and hemodialysis. Jornal brasileiro de nefrologia..

[30] Tannor EK, Archer E, Kapembwa K (2017). Quality of life in patients on chronic dialysis in South Africa: a comparative mixed methods study. BMC Nephrol.

[31] Theofilou P (2011). Quality of life in patients undergoing hemodialysis or peritoneal dialysis treatment. J Clin Med Res.

[32] Turkmen K, Yazici R, Solak Y (2012). Health-related quality of life, sleep quality, and depression in peritoneal dialysis and hemodialysis patients. Hemodial Int.

[33] Watanabe Y, Ohno Y, Inoue T (2014). Home hemodialysis and conventional in-center hemodialysis in Japan: a comparison of health-related quality of life. Hemodial Int.

[34] Wright LS, Wilson L (2015). Quality of life and self-efficacy in three Dialysis modalities: Incenter hemodialysis, home hemodialysis, and home peritoneal Dialysis. Nephrol Nurs J.

[35] Wu F, Cui L, Gao X (2013). Quality of life in peritoneal and hemodialysis patients in China. Ren Fail.

[36] Yang F, Griva K, Lau T (2015). Health-related quality of life of Asian patients with end-stage renal disease (ESRD) in Singapore. Qual Life Res.

[37] Ying SC, Krishnan M (2014). Interpretation of quality of life outcomes amongst end stage renal disease patients in selected hospitals of Malaysia. Int J Pharm Sci Res.

[38] Yongsiri S, Thammakumpee J, Prongnamchai S (2014). The association between bioimpedance analysis and quality of life in pre-dialysis stage 5 chronic kidney disease, hemodialysis and peritoneal dialysis patients. J Med Assoc Thail.

[39] Ware JE, Kosinski MM, Keller SD (1996). A 12-item short-form health survey: construction of scales and preliminary tests of reliability and validity. Med Care.

[40] Weisbord SD, Fried LF, Arnold RM (2004). Development of a symptom assessment instrument for chronic hemodialysis patients: the Dialysis symptom index. J Pain Symptom Manag.

[41] van der Willik Esmee, Leegte Martijn, van Ittersum Frans, Prantl Karen, Bart Hans, Dekker Friedo, Hemmelder Marc (2018). FP647FIRST DUTCH REGISTRY OF PATIENT-REPORTED OUTCOME MEASURES (PROMS) HAS A LOW RESPONSE IN DIALYSIS PATIENTS. Nephrology Dialysis Transplantation.

[42] Van der Willik EM, Meuleman Y, Prantl K, van Rijn G, Bos WJW, van Ittersum FJ, Bart HAJ, Hemmelder MH, Dekker FW. Patient-reported outcome measures: selection of a valid questionnaire for routine symptom assessment in patients with advanced chronic kidney disease – a four-phase mixed methods study. BMC Nephrology. 2019. Accepted. 10.1186/s12882-019-1521-9. 10.1186/s12882-019-1521-9 PMC6720373 31477039

[43] Ware JE (1997). SF-36 Health Survey. Manual and Interpretation Guide.

[44] Wyld M, Morton RL, Hayen A (2012). A systematic review and meta-analysis of utility-based quality of life in chronic kidney disease treatments. PLOS..

[45] Gandek B, Ware JE, Aaronson NK (1998). Cross-validation of item selection and scoring for the SF-12 health survey in nine countries: results from the IQOLA project. J Clin Epidemiol.

[46] Loosman WL, Hoekstra T, van Dijk S (2015). Short-form 12 or short-form 36 to measure quality-of-life changes in dialysis patients?. Nephrol Dial Transplant.

[47] World Health Organization (2014). ICD-10. Internationale statistische classificatie van ziekten en met gezondheid verband houdende problemen, tiende revisie.

[48] ERA-EDTA codes mortality. https://www.nefrovisie.nl/wp-content/uploads/2016/08/renine_codes_doodsoorzaak.pdf. Accessed 2 May 2019.

[49] Bouwmans C, Krol M, Severens H (2015). The iMTA productivity cost questionnaire a standardized instrument for measuring and valuing health-related productivity losses. Value Health.

[50] Institute for Medical Technology Assessment, Productivity and Health Research Group. Handleiding iMTA Medical Cost Questionnaire (iMCQ): iMTA, Erasmus University Rotterdam; 2018. https://www.imta.nl/questionnaires/. Accessed 7 June 2016.

[51] Hakkaart-van Roijen L, Van der Linden N, Bouwmans CAM (2015). Kostenhandleiding: Methodologie van kostenonderzoek en referentieprijzen voor economische evaluaties in de gezondheidszorg.

[52] Versteegh MM, Vermeulen KM, Evers SMAA (2016). Dutch tariff for the five-level version of EQ-5D. Value Health.

[53] Brooks R, Group E (1996). EuroQol: the current state of play. Health Policy.

[54] Eikelenboom N, van Lieshout J, Wensing M (2013). Implementation of personalized self-management support using the self-management screening questionnaire SeMaS; a study protocol for a cluster randomized trial. Trials..

[55] Eikelenboom N, Smeele I, Faber M (2015). Validation of self-management screening (SeMaS), a tool to facilitate personalised counselling and support of patients with chronic diseases. BMC Fam Pract.

[56] Lan PG, Clayton PA, Johnson DW (2016). Duration of hemodialysis following peritoneal dialysis cessation in Australia and New Zealand: proposal for a standardized definition of technique failure. Perit Dial Int.

[57] Hall YN, Larive B, Painter P (2012). Effects of six versus three times per week hemodialysis on physical performance, health, and functioning: frequent hemodialysis network (FHN) randomized trials. Clin J Am Soc Nephrol.

[58] Lowrie EG, Curtin RB, LePain N (2003). Medical outcomes study short form-36: a consistent and powerful predictor of morbidity and mortality in dialysis patients. Am J Kidney Dis.

[59] Korevaar JC, Jansen MAM, Merkus MP (2000). Quality of life in predialysis end-stage renal disease patients at the initiation of dialysis therapy. Perit Dial Int.

[60] Dolan P (1997). Modeling valuations for EuroQol health states. Med Care.

[61] Walters SJ, Brazier JE (2005). Comparison of the minimally important difference for two health state utility measures: EQ-5D and SF-6D. Qual Life Res.

[62] Culleton BF, Walsh M, Klarenbach SW (2007). Effect of frequent nocturnal hemodialysis vs conventional hemodialysis on left ventricular mass and quality of life. JAMA..

[63] Jardine MJ, Gray NA, De Zoysa J (2015). Design and participant baseline characteristics of ‘a clinical trial of IntensiVE Dialysis': the ACTIVE Dialysis study. Nephrology..

[64] Korevaar JC, Feith GW, Dekker FW (2003). Effect of starting with hemodialysis compared with peritoneal dialysis in patients new on dialysis treatment: a randomized controlled trial. Kidney Int.

