# Supplementary material for: Dutch nOcturnal and hoME dialysis Study To Improve Clinical Outcomes (DOMESTICO): rationale and design
Source: BMC Nephrol. 2019 Sep 18;20:361. doi: 10.1186/s12882-019-1526-4 (PMC6751675; doi:10.1186/s12882-019-1526-4)
Supplement: Supplementary file 1 — Local ethics committees/IRBs DOMESTICO. This file contains a list of the 44 (out of 53) local ethics committees from which approval for DOMESTICO is obtained. (DOCX 17 kb) [file 12882_2019_1526_MOESM1_ESM.docx]

**Additional file 1.** Local ethics committees/IRBs DOMESTICO

Admiraal de Ruyter Hospital (ADRZ2018-016 DOMESTICOpro)

Alrijne Hospital (19.172yw.tk)

Amphia Hospital (1620)

Bernhoven (T2018-03-02)

Bravis Hospital (PAC-2018-25-DOMESTICO studie)

Canisius-Wilhelmina Hospital (113-2018)

Catharina Hospital (CZE-2018.25)

Clinical Trial Center Maastricht (Maastricht UMC+) (181041)

Deventer Hospital (ME 18-30)

Dianet (Amsterdam and Utrecht)

Diapriva Dialysis Center

Elisabeth-TweeSteden Hospital

Elyse Clinics

Erasmus Medical Center (MEC-2018-1419)

Flevohospital (F18/24)

Franciscus Gasthuis & Vlietland (2018-073/T110)

Gelre Hospitals (18.22)

Haaglanden Medical Center (2018-023)

HagaHospital (T18-114)

Hospital Gelderse Vallei (1901-002)

Isala (190101)

Jeroen Bosch Hospital (2018.17.01)

Laurentius Hospital (C09064-MW/LH)

Maasstad Academie (L2018072)

Martini Hospital (2018-020)

Máxima Medical Center (L18.140)

Meander Medical Center (Niercentrum Midden Nederland) (TWO 18-63)

Medical Center Leeuwarden (COV 305)

Northwest Clinics (L018-034)

OLVG Amsterdam (WO 18.041)

Radboudumc Technology Center Clinical studies

Reinier de Graaf Gasthuis (18-418)

Rode Kruis Hospital (Dialysiscenter Beverwijk) (18.006/dw)

Slingeland Hospital (OND.2018.019 DOMESTICO)

Spaarne Gasthuis (2018.97)

St. Antonius Hospital (L18.035)

Treant Zorggroep (19119)

University Medical Center Groningen (local approval for University Medical Center Groningen and Dialysis Center Groningen) (2018/693)

University Medical Center Utrecht (18-096/R)

Viecuri Medical Center (394)

VU University Medical Center (2017.491)

Zaans Medical Center

Zuyderland (Z2018097)

For any future centres which will be included in our study, additional ethical approval will be sought.
